# Molecular Characterization of African Swine Fever Virus Isolates in Estonia in 2014–2019

**DOI:** 10.3390/pathogens9070582

**Published:** 2020-07-17

**Authors:** Annika Vilem, Imbi Nurmoja, Tarmo Niine, Taavi Riit, Raquel Nieto, Arvo Viltrop, Carmina Gallardo

**Affiliations:** 1 Institute of Veterinary Medicine and Animal Sciences, Estonian University of Life Sciences, Kreutzwaldi 62, 51006 Tartu, Estonia; Imbi.Nurmoja@vetlab.ee (I.N.); Tarmo.Niine@emu.ee (T.N.); arvo.viltrop@emu.ee (A.V.); 2 Veterinary and Food Laboratory, Kreutzwaldi 30, 51006 Tartu, Estonia; taaviriit@hotmail.com; 3 European Union Reference Laboratory for ASF, INIA-CISA, 28130 Valdeolmos, Spain; nieto.raquel@inia.es (R.N.); gallardo@inia.es (C.G.)

**Keywords:** African swine fever, CVR, genotyping, molecular characterization

## Abstract

After the extensive spread of the African swine fever virus (ASFV) genotype II in Eastern Europe, the first case of African swine fever (ASF) in Estonia was diagnosed in September 2014. By the end of 2019, 3971 ASFV-positive wild boars were found, and 27 domestic pig outbreaks were reported. A selection of ASFV isolates from wild boar and domestic pigs (during the period of September 2014–2019) was molecularly characterized using standardized genotyping procedures. One of the proven markers to characterize this virus is the central variable region (CVR) within the *B602L* gene. In summer 2015, a new ASFV genotype II CVR variant 2 (GII-CVR2) was confirmed in Estonia. The results suggest that the GII-CVR2 variant was only confirmed in wild boar from a limited area in southern Estonia in 2015 and 2016. In addition to GII-CVR2, a single nucleotide polymorphism (SNP) that resulted in amino acid change was identified within the genotype II CVR variant 1 (GII-CVR1). The GII-CVR1/SNP1 strain was isolated in Estonia in November 2016. Additional GII-CVR1/SNP1 cases were confirmed in two neighbouring counties, as well as in one outbreak farm in June 2017. Based on the available data, no GII-CVR2 and GII-CVR1/SNP1 have been reported by other affected European countries. The spread of variant strains in Estonia has been limited over time, and restricted to a relatively small area.

## 1. Introduction

African swine fever (ASF) is a viral disease of swine that can lead to high mortality in domestic pigs and wild boar *(Sus scrofa)* while being asymptomatic in wild suid reservoir hosts in Africa [1]. ASF causes important economic damage due to mortality and production losses. The main economic influence of this virus comes from its radical control measures, as well as the ban on international trade of live animals and meat products [2,3]. The causative agent of ASF is a large enveloped double-stranded DNA virus, the member of the family *Asfarviridae* within the genus *Asfivirus*. The length of the African swine fever virus (ASFV) genome can vary remarkably from 170 to 194 kbp, and the number of genes can vary from 150 to 167, depending on the isolate [4,5,6,7]. The DNA molecule of the virus encodes between 151 and 167 open reading frames (ORFs) on both strands [1,8]. The gain or loss of ORFs from the multigene families (MGF) encoded by the virus is the primary reason behind the differences in the virus’s genome length and gene number [5,6,9]. A large number of highly variable multigene families in the genome of ASFV is one of the biggest challenges for understanding the functionality of the virus [5].

Endemic in more than 20 sub-Saharan African countries [10], as well as in Sardinia since the last century [11], ASF arrived at a Black Sea harbor in Georgia in 2007 [12]. The disease spread quickly west and northwards, reaching the European Union (EU) in 2014. Currently, ASFV is present in ten EU countries, including Lithuania, Poland, Latvia, Estonia, Romania, Bulgaria, Hungary, Belgium, Slovakia, and Greece, which was recently affected in 2020 [13]. Outbreaks have also occurred in Asia since August 2018, when China declared the presence of ASFV in domestic pigs [14]. The latter constitutes one of the most important jumps of the disease thus far. The continuous spread to other Asian countries, and recently to Oceania [15], makes controlling the spread of the virus even more difficult. Early detection and coordinated countermeasures of ASFV are urgently needed; however, for such measures to be effective, information regarding the disease’s dynamics must be determined. Thus, molecular epidemiology has become an essential part of the epidemiological investigation.

The current genotyping of ASFV strains is based on partial nucleotide sequencing of the *B646L* gene, which encodes the major capsid p72 protein [16,17]. In total, 24 genotypes of ASFV have been determined worldwide [16,18,19]. All genotypes are present in Africa, but only two of them (genotype I and II) have been found on other continents [16,20,21]. In Europe, genotype I has circulated since 1978 on the Italian island of Sardinia. Genotype II began to circulate in large areas of eastern Europe in 2007 [3,12,22], spreading consistently westwards and eastwards, affecting new countries and territories in Europe and Asia [23,24,25]. Although the partial p72 sequencing has proved useful for placing the viruses within one of the defined 24 genotypes, a higher resolution is needed for an in-depth analysis. Several studies have described the suitability of the sequencing of tandem repeat sequences (TRSs) located in the central variable region (CVR) within the *B602L*-gene to distinguish between closely related ASF isolates belonging to a single genotype [17,26,27,28]. Until this study only one CVR variant (GII-CVR1, the “Georgia type variant”) was described in ASFV genotype II, characterized by the 10 amino acid TRS [23,29] Nevertheless, this region was selected for a deeper molecular characterization of the selected Estonian ASFV isolates collected since September 2014, when the first case of ASF was diagnosed in a wild boar found dead in southern Estonia near the Latvian border. A few weeks later, an ASFV positive wild boar was found in the northeast of Estonia, near the border with the Russian Federation and 200 km away from the first case of ASF in the south [30,31]. Based on further molecular and epidemiological analyses, this finding was not considered to be epidemiologically connected with the findings in the south [30,31,32]. In the following three years, there was extensive spread of the ASFV in the wild boar population, and by the end of 2017, wild boar from 14 out of 15 counties were affected [33,34]. The first domestic pig outbreak occurred in July 2015. In total, 27 outbreaks were reported in domestic pig farms, and 42,476 pigs were culled in outbreak farms during the period of 2015–2017 [33]. 

Starting from the beginning of the ASF epidemics in 2014, virus strains have been collected systematically from both wild boar and domestic pigs to describe the genetic variability of ASFV strains circulating in Estonia and to investigate the distribution of genetic variants of the virus. A selection of the results from these investigations is presented in this paper.

## 2. Materials and Methods

### 2.1. Sampling

The sampling scheme for ASF surveillance among wild boar and domestic pig populations was done in accordance with the Estonian ASF control program, compiled by the veterinary and food authority. This program included the sampling of wild boar found dead and hunted from affected and non-affected areas, as well as domestic pigs in outbreak herds and non-affected herds for various surveillance purposes [31,34]. From wild boar, blood samples for ASFV and antibody detection were collected immediately after the boar were hunted. From animals found dead, organ samples were collected for ASFV detection by official veterinarians [31]. Blood samples were collected from domestic pigs for virus and antibody detection. Organ samples were collected from dead or culled animals for virus detection only. All samples were submitted for analysis to the Veterinary and Food Laboratory, which is the National Reference Laboratory (NRL) for ASF. The samples were stored at +4 °C until analysis, and only a small number of organ samples were stored at −20 °C before transport to the laboratory. 

### 2.2. Selection of Isolates for Molecular Characterization 

Three hundred and ninety-six (*n* = 396) polymerase chain reaction (PCR) positive samples were selected for further molecular characterization. The NRL regularly sent a selection of samples to the European Union Reference Laboratory (EURL) of ASF (INIA-CISA, Valdeolmos, Spain) for virus isolation and further biological and molecular characterization studies. The selection of samples from wild boar was based on the principles of purposive and judgment sampling. The first aim was to represent the entire affected area; thus, samples were selected based on the spatial and temporal distribution of positive virus findings. Secondly, wild boar cases in the proximity of domestic outbreaks were selected for further analyses. Third, the surrounding areas were more intensively screened to find genetic variants of the virus and define the distribution of variant strains. Finally, in the later stages of the epidemic (in 2018 and 2019), the target was to characterize new isolates collected from the areas where ASFV PCR-positive wild boar were not found for longer periods. The isolates from all domestic pig outbreak farms were subject to further molecular characterization (*n* = 48). The record of isolates selected for molecular characterization per county and year is presented in Table 1.

### 2.3. Detection of the Genome of ASFV

DNA was extracted from serum or tissue (spleen, kidney, or bone marrow) samples using the QiaAmp Cador Pathogen Mini kit or Cador Pathogen HT kit (Qiagen) and an automated extraction system QiaCube or QiaCube HT (Qiagen) according to the manufacturer’s instructions, with starting material of 200 µL. A 10% tissue suspension was performed with nuclease-free water using a Precellys24 Tissue Homogenizer, and the tubes were filled with ceramic beads (Bertin Technologies) prior to viral DNA extraction. For real-time PCR, the primers and TaqMan probe were used for ASFV p72 gene detection, as presented by Tignon et al. 2011 [35]. For endogenous control of the assay, swine beta-actin (ACTB) gene detection was included in the analysis using the primers and probe targeting the 114 bp-region of the gene [36]. Real-time PCR was carried out using a commercially available 5xHOT FIREPol Probe qPCR Mix kit (Solis BioDyne) with a total volume of 20 µL. Briefly, 7 µL of DNase RNase free water, 4 µL 5× HOT FIREPol Probe qPCR Mix, and 0.8 µL each of forward and reverse primers targeting the ASF p72 and swine beta-actin gene in a final concentration of 0.4 µM and 0.4 µL each for the probe in a final concentration of 0.2 µM were pooled together as a master mix. Finally, a 5 µL of aliquot of DNA extracted from the sample was added to 15 µL of the PCR master mix. The cycling protocol was as follows: One cycle of 95 °C for 15 min, followed by 45 cycles consisting of denaturation for 20 sec at 95 °C, and annealing for 1 min at 60 °C. The threshold cycle (Ct) values less than 37 were considered positive.

### 2.4. B602L Gene Amplification and Sequencing

Two hundred-and-forty-four (202 from wild boar and 42 from domestic pig) isolates were sequenced by the EURL for ASF (INIA-CISA) according to the protocol previously described [27]. Additionally, at the NRL, *B602L* gene amplification of 152 ASFV DNA isolates was performed (146 from wild boar and 6 from domestic pigs) using the protocol provided by INIA-CISA with minor modifications. Briefly, the final concentration of the primers was decreased to 0.3 µM, and the annealing time under PCR cycling conditions was decreased to 30 sec. Further sequencing was performed at the University of Tartu (Estonia), Institute of Genomics. Nucleotide sequences were edited and analyzed using the MEGA7.0.26 software with the ClustalW alignment [37]. All sequences were aligned with the Georgia2007/1 reference sequence (GenBank accession number FR682468.1) [38].

Aligned nucleotide sequences were translated into amino acids using the MEGA7.0.26 software to determine the changes in amino acid sequences compared to the reference mentioned above [37]. The nucleotide sequences of the Estonian ASFVs belonging to GII-CVR2 and GII-CVR1/SNP1 variants were deposited in GenBank (Accession numbers from MT647527 to MT647567, Table S1). In addition, all the Estonia sequences, including the GII-CVR1 variant (Georgia type variant) generated in this study, are available on request at the EURL for ASF at http://asf-referencelab.info/asf/en/ (Table S1).

### 2.5. Phylogenetic Analysis

Aligned nucleotide sequences of the CVR were used to calculate the phylogenetic tree with the Maximum Likelihood (ML) method using the Jukes-Cantor model with 500 bootstrap replicates (implemented in MEGA7.0.26 software). Isolates from the EURL sequence database were used to generate a comparable topology of the tree. 

### 2.6. Mapping Software and Spatial Autocorrelation

The QGIS Geographic Information System [39] software (version 3.4.3-Madeira) was used to visually analyze the possible spatial clustering of different ASFV CVR variants. A map of Estonia was obtained from the Estonian land board [40] website: “Map of municipal counties before public administration reform”. The map’s coordinate reference system (CRS) was set to EPSG:3301—the Estonian Coordinate System of 1997. All the municipality borders were removed, as they were not needed for the present analysis.

To visually assess the spatial clustering of ASFV CVR variants, the Heatmap (Kernel Density Estimation) algorithm in the QGIS software was used with the radius set to 7000 m. 

Moran’s global index of spatial autocorrelation I was calculated for ASFV locations using the STATA 14.0 software (StataCorp LP, College Station, USA) packages Spmap [41] and Spatwmat [42]. The distance band was set to 7000 m with an inverse distance weight matrix.

## 3. Results

### 3.1. B602L Gene Amplification and Sequencing 

Amplification of the *B602L* variable region was achieved for 335 out of the 348 wild boar, and all 48 domestic pigs tested. Amplicons of 400 bp were generated from all domestic pigs and from 318 wild boar, whereas 17 wild boar samples had an amplicon of approximately 350 bp. 

**Figure 1:**
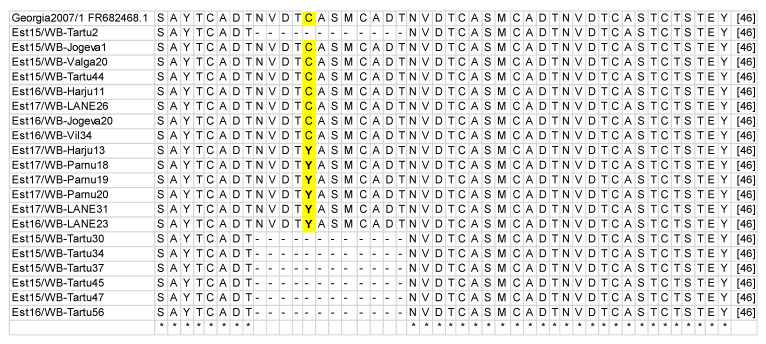
Amino acid *(aa)* alignment of Estonian strains. The GII-CVR1/SNP1 strain has an *aa* change marked with yellow (Y instead of C). Sequences with the deletion of three *aa* tandem repeats are determined as GII-CVR2.

Further nucleotide and amino acid sequence analyses revealed three different variants co-circulating in the country, two of them within the ASFV GII-CVR1, according to the nomenclature of Gallardo et al. [29,43]. The CVR sequences of 300 wild boar and 42 domestic pigs that yielded an amplicon of 400 bp were 100% homologous to the reference sequence of Georgia 2007/1 (GenBank accession number FR682468.1) [38], and were classified as ASFV genotype II CVR variant 1 (GII-CVR1). Sequence analysis of the remaining 18 wild boar and six domestic pigs revealed a single nucleotide polymorphism (SNP) within the CVR variant 1 (GII-CVR1/SNP1), where guanine (G) was replaced with adenine (A). This transition also resulted in an amino acid change, where cysteine (C) was replaced with tyrosine (Y) (Table 2, Figure 1). The size difference found in the 17 wild boar samples, which resulted in amplicons of approximately 350 bp, was due to the deletions of three amino acid TRSs (types NVDT, CASM, and CADT) producing a total of seven TRSs instead the 10 aa tetramer repeats present within the GII-CVR1 variant. The number of samples, amplicon sizes, and differences are shown in Table 2. 

A visualization of the amino acid changes in the GII-CVR1/SNP1 strain and the deletion of the three amino acid tandem repeats in GII-CVR2 are shown in Figure 1.

### 3.2. Phylogenetic Analysis

**Figure 2:**
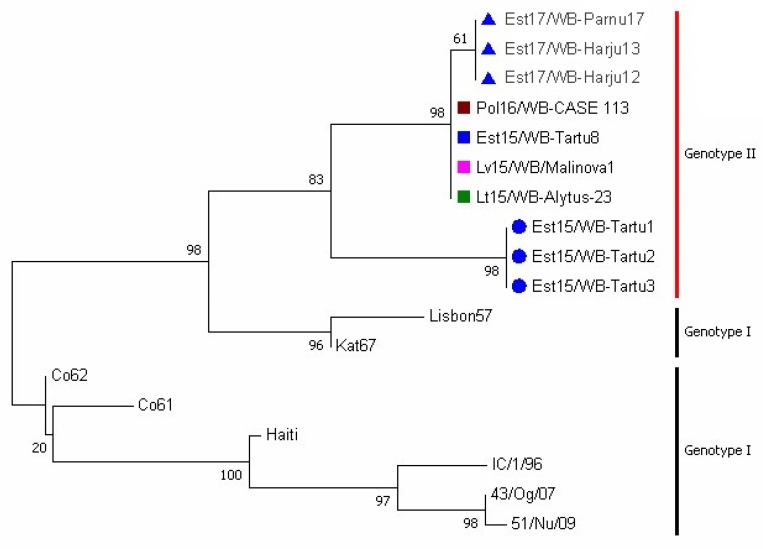
Phylogenetic tree of the CVR sequence within the B602L gene. ● GII-CVR2 sequence determined in Estonia; □ GII-CVR1 sequences from Estonia (blue), Poland (red), Latvia (rose), and Lithuania (green); ▲ GII-CVR1-SNP1 sequence determined in Estonia. Numbers at the nodes represent the percentage of 500 bootstrap replicates.

A phylogenetic tree of representative CVR sequences within the *B602L* gene is presented in Figure 2. The Estonian ASFV GII-CVR1 and GII-CVR2 variants are clustered in separate branches and share a common ancestor. The GII-CVR1/SNP1 variant evolved from GII-CVR1, and its branch length indicates a minor deviation from its ancestor (GII-CVR1). The GII-CVR1 variant strains isolated from the Baltic States and Poland are 100% homologous and cluster together as expected. A portion of the genotype I isolates was added to the tree construction to visualize the relationships between the CVRs of the two genotypes.

### 3.3. Geographic Distribution of CVR Variants

**Figure 3:**
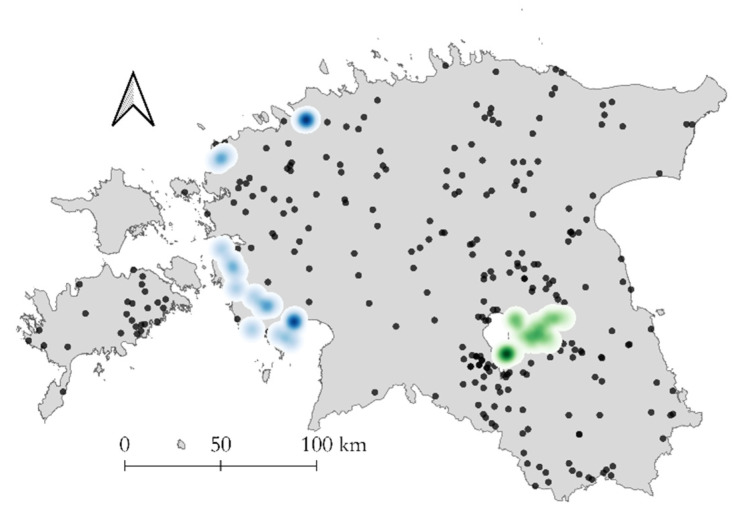
Spatial distribution of characterized African swine fever virus (ASFV) isolates and clustering of the variant strains from the period of 2014–2019 in Estonia. The spread of GII-CVR1 is marked with black dots. Both GII-CVR1/SNP1 (blue) and GII-CVR2 (green) are presented as Kernel density estimates with the radius set to 7000 m.

A significant positive global spatial autocorrelation in the distribution of the CVR variants was detected. The Moran’s I value was 0.715 (z = 6.197; *p* < 0.001). The spatial autocorrelation can also visually be seen in Figure 3. 

GII-CVR2 circulated only in the wild boar population in one county (Tartu). In total, four municipalities were affected by GII-CVR2. The first finding of the new GII-CVR2 was detected from wild boar found dead in the middle of July 2015 in the municipality of Rannu. At the end of July, GII-CVR2 was found in the neighboring Konguta municipality. In autumn 2015, the new variant spread further to two other neighboring municipalities, Tähtvere and Nõo. The co-circulation of both genetic variants (GII-CVR1 and GII-CVR2) was observed at the same time in this area. The last finding of GII-CVR2 dates to the end of March 2016. All findings of GII-CVR2 are summarized in Table 3. Among all the GII-CVR2 isolates, 14 samples out of 17 originated from wild boars found dead.

The GII-CVR1/SNP1 variant strain was first detected in a wild boar found dead in Lääne county (municipality Hanila) at the end of November 2016. In total, three counties were affected by the CVR1/SNP1 strain, and 18 wild boars were determined to be CVR1/SNP1 positive. The CVR1/SNP1 findings among the wild boar are summarized in Table 4. Six domestic pig samples determined to be CVR1/SNP1 positive originated from the same outbreak farm in the municipality of Audru.

## 4. Discussion 

The molecular surveillance of ASFV is an integral part of the disease intervention activities in affected countries. Most published studies use the molecular characterization of vp72 or/and variable regions containing an array of the tandem repeat sequences (TRS) for genotyping and subgrouping closely related isolates [16,17,21,26,29,44]. Recent investigations of the genetic diversity of ASFV genotype II isolates have been based on sequencing the intergenic region (IGR) between the I73R and I329L genes [22,25,45,46]. A study performed at the EURL (Valdeolmos, Spain) analyzed the nucleotide sequences of the IGR of 232 isolates collected in Estonia (2014–2018), but did not reveal any genetic modifications between them [23]. All isolates had the same additional tandem-repeat sequence (TATATAGGAA) representative of the intergenic region (IGR) 2 variant [29], and could not be separated into subgroups. 

A higher phylogenetic resolution between closely related ASFV genotype isolates in Europe and in Africa was achieved by analyzing the amino-acid TRSs located in the CVR within the *B602L* gene [26,27,47]. In this study, a comparative analysis of PCR CVR size fragments enabled the researchers to identify three different CVR variants of ASFV that have circulated in certain regions of Estonia since 2014. The selection of the samples was initially based on representing all affected areas. Nevertheless, the sample choice was mostly concentrated in the county of Tartu, where the first GII-CVR2 finding was detected, as well as in the two neighboring counties. The same strategy was used for samples originating from the county of Lääne, where the first GII-CVR1/SNP1 variant was detected. Therefore, the sampling scheme was not completely random, as the aforementioned areas were investigated more intensively. In total, 11.6% of all ASFV positive samples collected from wild boar were selected for further CVR sequence analyses. Sequencing of the 13 samples likely failed, due to a weak viral load or insufficient sample quality. The conventional PCR (cPCR) used in prior *B602L* gene sequencing is less sensitive than the real-time PCR used in ASFV genome detection. ASFV positive samples with ct values over 34 are likely to fail in cPCR. Nevertheless, the method used in this study was slightly modified (see material and methods) for adaptation to our laboratory equipment and reagents to obtain optimal results. The real-time PCR method used in ASFV genome detection was also changed by adjusting its protocol to the PCR kit available in the laboratory. Despite the changes in the PCR reagents, the in-house validation of this method and excellent annual participation in interlaboratory comparison tests, organized by the EURL for ASF, confirm that this method is fit for its intended purpose. 

CVR amplification using the cPCR of wild boar samples allowed us to identify, on July 2015, a size variation in the amplicon obtained from two wild boar samples collected in Tartumaa. Sequence analysis revealed that this difference in size was due to the deletion of three amino acid tetramer repeats compared to the GII-CVR1 “Georgia type” sequences circulating in Europe since 2007 [12,23,29]. These isolates were classified as a new variant named the GII-CVR2 variant. Since the first identification of the GII-CVR2 variant, the number of investigations has noticeably increased in the region and in the two neighboring counties (Valga and Viljandi) to further clarify the spread of the novel variant strain. From the results obtained, we observed that the spread of the GII-CVR2 strain occurred over a relatively short span of time (from July 2015 to March 2016), and only in four municipalities of one county (Tartu)—where both GII-CVR variants co-circulated at the same time. Similar CVR size variation was previously described in Europe (in Sardinia), where genotype I predominates. The changes in the CVR region occurred a few decades after the virus entered Sardinia, placing the isolates into two clusters depending on their temporal distribution [48,49]. Investigations in Estonia revealed that the amino acid deletion within genotype II occurred eight years after the virus entered into Georgia in 2007. This deletion could be related to a spontaneous mutation caused by the maintenance of ASFV within the wild boar population in the county of Tartu, since this phenomenon was not found in other affected areas in Estonia or in Europe [23]. However, since new variants in the ASF genome are difficult to locate if the number of investigations is low, the introduction of GII-CVR2 to Estonia from other affected areas cannot be excluded completely. 

Based on the results obtained, the geographical evolution indicates the extinction of new genetic variants over time. The animal trial conducted using one of these GII-CVR2 isolates (Est15/WB-Tartu14) classified the ASFV strain as moderately virulent [22]. We, therefore, hypothesize that the disappearance of GII-CVR2 could be connected to the reduced virulence of the strain. However, this animal trial conducted with the Estonian GII-CVR2 (Est15/WB-Tartu14) isolate also included an Estonian GII-CVR1 strain (Est15/WB-Valga6) in parallel [22]. Clinical signs of the disease observed in this study were acute, subacute, and chronic; such signs are usually connected to viral strains of moderate virulence [50]. This was the first ASFV genotype II virus strain to show moderate virulence in Europe. Until this study, subacute and chronic causes of the disease, as well as moderate virulence, were only connected with genotype I in Europe [22,51,52]. Irrespective of the variant strain used in the experiment, reduced virulence was observed [22]. Therefore, the deletion of three amino acid tetramer repeats in the *B602L* gene described in this study cannot be directly associated with reduced virulence, and the genes connected with moderately virulent strains need further research. We conclude that there is a possibility of domestic pigs and wild boar to develop chronic forms of this disease, a phenomenon common to ASFV strains with reduced virulence [22,53,54]. Reduced virulence ASFV genotype II isolates were also described in two additional cases in Europe, while attenuated strains of the ASFV genotype II were found in Estonia [32] and Latvia [55]. By the time, genotype II ASFV circulating in Europe was known to be highly virulent and led to a 90–100% mortality in domestic pigs and European wild boar under experimental conditions [8,53,54,56]. 

The GII-CVR1/SNP1 detected in the western and northern parts of Estonia is a non-synonymous mutation that alters the amino acid sequence, resulting in tyrosine coding instead of cysteine. The relevance of this change remains unknown and needs further investigation. Additional SNP variants within the genotype II CVR1 sequence have been reported in Poland and Lithuania, where GII-CVR1/SNP2 and GII-CVR1/SNP3 are circulating, respectively [23]. However, compared to GII-CVR1/SNP1, the SNP variants detected in Poland and Lithuania are in a different position and do not alter the amino acid sequence [43]. Using the whole genome sequencing approach, SNPs have also been described in other gene regions [24,57], and are the most common types of genetic variations in the ASFV genome. Therefore, there is a possibility that SNP1 or other SNPs are also present in the southern and eastern areas of Estonia, but because positive ASF cases have decreased since the end of 2016 in the mentioned areas, these mutations may remain undetected. 

Compared to GII-CVR2, the spread of the GII-CVR1/SNP1 strain in Estonia took a longer period of time (from November 2016 to December 2017) and covered a larger area (three counties). The epidemic of ASF was severe in the western part of Estonia at the time when GII-CVR1/SNP1 was identified in the county of Lääne. Therefore, the spread of this strain to neighboring counties was likely during this period. Due to the decreased wild boar population, the number of positive ASF cases in wild boar have decreased significantly since the end of 2017 [34], which may explain the lack of GII-CVR1/SNP1 findings after this period. 

Notably, GII-CVR1/SNP1 was also responsible for a domestic pig outbreak that occurred in the county of Pärnu in the middle of June 2017. A temporal investigation revealed the circulation of the GII-CVR1/SNP1 strain in the wild boar population in the same village only a few weeks before the outbreak. This finding agrees with the conclusions of another Estonian study that argued the presence of ASFV in wild boar populations is the main risk for domestic pig farms becoming infected [33].

In conclusion, three changes were identified in the *B602L* gene of ASFV isolated from the Estonian wild boar population, with the GII-CVR1 “Georgia type” variant being predominant. The GII-CVR2 variant remained only in a limited area and disappeared after the end of March 2016, whereas the GII-CVR1/SNP1 variant was no longer detected after December 2017. These data suggest that there is a predominant variant causing the outbreaks in Estonia, as no significant genetic changes have occurred in the regions evaluated over the five-year period. Although analysis of the CVR has been widely used to distinguish between closely related ASF isolates in Africa [27,28] and Europe [29], the low CVR genetic variability necessitates further research into alternative and more informative gene regions to clarify the relevant intra genotype relationships. Recent studies on the molecular evolution of genotype II EU strains revealed the presence of five different variants circulating in the EU when sequencing the IGR between the MGF505 9R and 10R genes [Gallardo personal communication 2019]. An extensive analysis of Estonian ASFV isolates using this genetic marker could help clarify questions regarding the epidemiology of ASF in Estonia.

## Figures and Tables

**Table 1:** The record of African swine fever virus (ASFV) polymerase chain reaction (PCR)-positive samples and performed sequencing analyses for central variable region (CVR) variant determination among wild boars in Estonia during the period 2014–2019.

Year	2014		2015		2016		2017		2018		2019		CVR% *
County	PCR	CVR	PCR	CVR	PCR	CVR	PCR	CVR	PCR	CVR	PCR	CVR	
Harju	0	0	0	0	35	6	66	12	17	2	0	0	16.9
Ida-Viru	4	3	10	2	22	3	3	1	4	4	4	2	31.9
Jõgeva	0	0	55	6	146	14	0	0	1	0	0	0	9.9
Järva	0	0	98	9	95	5	0	0	0	0	0	0	7.3
Lääne	0	0	0	0	57	13	97	16	10	2	1	0	18.8
L-Viru	0	0	82	6	171	9	42	4	0	0	0	0	6.4
Põlva	0	0	227	6	166	2	9	4	3	1	0	0	3.2
Pärnu	0	0	23	3	76	6	68	13	5	0	0	0	12.8
Rapla	0	0	6	2	181	6	51	7	1	0	0	0	6.3
Saare	0	0	0	0	90	8	236	16	15	1	1	0	7.3
Tartu	0	0	117	38	151	26	13	6	1	1	0	0	25.2
Valga	13	6	113	17	10	3	0	0	0	0	0	0	19.1
Viljandi	47	9	166	22	37	6	3	1	0	0	0	0	15.0
Võru	9	5	108	11	40	1	3	2	1	0	0	0	11.8
**TOTAL**	**73**	**25**	**1005**	**122**	**1277**	**108**	**591**	**82**	**58**	**11**	**6**	**2**	**11.6**

* % of samples among PCR positive wild boar selected for CVR determination.

**Table 2:** Number of samples, amplicon sizes, and differences revealed in the sequencing of the central variable region (CVR) within *B602L* gene.

Size of an Amplicon (bp)	*No* of WB ^1^	*No* of DP ^2^	No of aa * Tetrameric Repeats	Difference From Reference Strain	CVR Variant
400	300	42	10	no	CVR1
400	18	6	10	G instead of A resulted in *aa* change Y instead of C	CVR1/SNP1
~350	17	0	7	deletion of 3 *aa* tetramer repeats CASMCADTNVDT	CVR2

^1^ Wild Boar. ^2^ Domestic pig. * amino acid.

**Table 3:** GII-CVR2 findings in four rural municipalities in Tartu county in 2015 and 2016.

			Number of Findings		
Municipality	First Finding	Last Finding	2015	2016	Total
Rannu	17th of July 2015	22nd of Feb 2016	5	2	7
Konguta	29th of July 2015	6th of Nov 2015	4	0	4
Tähtvere	29th of Sept 2015	29th of Mar 2016	2	1	3
Nõo	14th of Oct 2015	15th of Mar 2016	2	1	3
		**Total**	**13**	**4**	**17**

**Table 4:** GII-CVR1/SNP1 findings among the wild boar in three counties in 2016 and 2017.

				No of Findings		
County	Municipality	First Finding	Last Finding	2016	2017	Total
Lääne	Hanila	30th of Nov 2016	24th of Jan 2017	2	1	3
	Nõva	10th of Aug 2017	10th of Aug 2017	0	1	1
	Noarootsi	12th of Dec 2017	12th of Dec 2017	0	1	1
Harju	Keila	5th of Jan 2017	4th of July 2017	0	3	3
Pärnu	Varbla	18th of Jan 2017	16th of June 2017	0	2	2
	Tõstamaa	20th of Feb 2017	12th of Dec 2017	0	6	6
	Audru	25th of May 2017	16th of June 2017	0	2	2
			**Total**	**2**	**16**	**18**

## Supplementary Materials

The following are available online at https://www.mdpi.com/2076-0817/9/7/582/s1, Table S1: African swine fever virus isolates selected to study CVR genetic variation.
