# Supplementary material for: Molecular Characterization of African Swine Fever Virus Isolates in Estonia in 2014–2019
Source: Pathogens. 2020 Jul 17;9(7):582. doi: 10.3390/pathogens9070582 (PMC7400522; doi:10.3390/pathogens9070582)
Supplement: Supplementary file 1 [file pathogens-09-00582-s001.pdf]

Table S1. African swine fever virus genotype II isolates from Estonia selected to study central variable region (CVR) genetic variation. **DP, domestic pig; EWB, European wild boar; *NRL, National Reference Laboratory

Isolate	NRL* registration number	Host Species**	Date outbreak	Municipality	County	CVR variant	GenBank Accession number
Est14/WB-Valga-1	TA1411593	EWB	02/09/2014	Hummuli	VALGA	GII-CVR1	
Est14/WB-VIL-1	TA1411917	EWB	09/09/2014	Tarvastu	VILJANDI	GII-CVR1	
Est14/WB-Valga-2	TA1412184	EWB	10/09/2014	Hummuli	VALGA	GII-CVR1	
Est14/WB-VIL-2	TA1412212	EWB	11/09/2014	Tarvastu	VILJANDI	GII-CVR1	
Est14/WB-VIL-3	TA1412213	EWB	11/09/2014	Tarvastu	VILJANDI	GII-CVR1	
Est14/WB-VIL-4	TA1412214	EWB	11/09/2014	Tarvastu	VILJANDI	GII-CVR1	
Est14/WB-IDA-1	TA1412317	EWB	14/09/2014	Lüganuse	IDA-VIRU	GII-CVR1	
Est14/WB-VORU-1	TA1415193_1	EWB	26/10/2014	Võrumaa	VORU	GII-CVR1	
Est14/WB-VORU-2	TA1415193_2	EWB	26/10/2014	Varstu	VORU	GII-CVR1	
Est14/WB-VORU-3	TA1415193_3	EWB	26/10/2014	Varstu	VORU	GII-CVR1	
Est14/WB-VORU-4	TA1415194	EWB	26/10/2014	Varstu	VORU	GII-CVR1	
Est15/WB-Valga-3	TA1500043	EWB	05/01/2015	Helme	VALGA	GII-CVR1	
Est15/WB-Valga-4	TA1500136	EWB	06/01/2015	Podrala	VALGA	GII-CVR1	
Est15/WB-Vil-5	TA1500130	EWB	06/01/2015	Tarvastu	VILJANDI	GII-CVR1	
Est15/WB-Vil-6	TA1500891	EWB	15/01/2015	Tarvastu	VILJANDI	GII-CVR1	
Est15/WB-Vil-7	TA1501143	EWB	20/01/2015	Tarvastu	VILJANDI	GII-CVR1	
Est15/WB-Vil-8	TA1501144	EWB	20/01/2015	Tarvastu	VILJANDI	GII-CVR1	
Est15/WB-Vil-9	TA1501120-1	EWB	20/01/2015	Tarvastu	VILJANDI	GII-CVR1	
Est15/WB-VORU-8	TA1504950-2	EWB	26/01/2015	Sõmerpalu	VORU	GII-CVR1	
Est15/WB-Vil-10	TA1501766	EWB	29/01/2015	Tarvastu	VILJANDI	GII-CVR1	
Est15/WB-Vil-11	TA1503048	EWB	29/01/2015	Tarvastu	VILJANDI	GII-CVR1	
Est15/WB-Vil-12	TA1503786	EWB	29/01/2015	Tarvastu	VILJANDI	GII-CVR1	
Est15/WB-Valga-5	TA1501895	EWB	02/02/2015	Podrala	VALGA	GII-CVR1	
Est15/WB-VORU-9	TA1505072-1	EWB	02/02/2015	Sõmerpalu	VORU	GII-CVR1	
Est15/WB-Vil-13	TA1502109	EWB	04/02/2015	Tarvastu	VILJANDI	GII-CVR1	
Est15/WB-Vil-14	TA1503048	EWB	16/02/2015	Tarvastu	VILJANDI	GII-CVR1	
Est15/WB-Valga-6	TA1503275	EWB	18/02/2015	Podrala	VALGA	GII-CVR1	
Est15/WB-Valga-7	TA1503644	EWB	25/02/2015	Podrala	VALGA	GII-CVR1	
Est15/WB-Vil-15	TA1503786	EWB	03/03/2015	Tarvastu	VILJANDI	GII-CVR1	

Est15/WB-Vil-16	TA1504600	EWB	03/03/2015	Tarvastu	VILJANDI	GII-CVR1	
Est15/WB-Vil-17	TA1501120-1	EWB	06/03/2015	Tarvastu	VILJANDI	GII-CVR1	
Est15/WB-Vil-18	TA1504355-1	EWB	06/03/2015	Tarvastu	VILJANDI	GII-CVR1	
Est15/WB-Valga-8	TA1504601	EWB	10/03/2015	Helme	VALGA	GII-CVR1	
Est15/WB-Vil-19	TA1504356-2	EWB	10/03/2015	Tarvastu	VILJANDI	GII-CVR1	
Est15/WB-Vil-20	TA1504600	EWB	17/03/2015	Tarvastu	VILJANDI	GII-CVR1	
Est15/WB-Vil-21	TA1504951-5	EWB	17/03/2015	Tarvastu	VILJANDI	GII-CVR1	
Est15/WB-VORU-5	TA1501501	EWB	17/03/2015	Varstu	VORU	GII-CVR1	
Est15/WB-VORU-6	TA1501932	EWB	17/03/2015	Varstu	VORU	GII-CVR1	
Est15/WB-VORU-7	TA1504949-1	EWB	18/03/2015	Varstu	VORU	GII-CVR1	
Est15/WB-Valga-9	TA1506915	EWB	19/04/2015	Puka	VALGA	GII-CVR1	
Est15/WB-Valga-10	TA1508417	EWB	18/05/2015	Puka	VALGA	GII-CVR1	
Est15/WB-Parnu1	TA1508481	EWB	18/05/2015	Vandra	PARNU	GII-CVR1	
Est15/WB-Valga-11	TA1509346	EWB	01/06/2015	Põdrala	VALGA	GII-CVR1	
Est15/WB-Vil22	TA1511227	EWB	07/07/2015	Viljandi	VILJANDI	GII-CVR1	
Est15/WB-IDA2	TA1511836	EWB	14/07/2015	Lüganuse	IDA-VIRU	GII-CVR1	
Est15/WB-Tartu1	TA1511809	EWB	17/07/2015	Rannu	TARTU	GII-CVR2	MT647527
Est15/DP-VALGA1	TA1511881	DP	18/07/2015	Taheva	Valga	GII-CVR1	
Est15/DP-Vil2	TA1511855	DP	18/07/2015	Kolga-Jaani	VILJANDI	GII-CVR1	
Est15/DP-Vil1	TA1511845	DP	18/07/2015	Viljandi	VILJANDI	GII-CVR1	
Est15/WB-Tartu3	TA1511843	EWB	19/07/2015	Rannu	TARTU	GII-CVR1	
Est15/WB-Tartu2	TA1511842	EWB	19/07/2015	Rannu	TARTU	GII-CVR2	MT647528
Est15/WB-VORU-10	TA1511920	EWB	21/07/2015	Võru	VORU	GII-CVR1	
Est15/WB-POLVA-1	TA1512135	EWB	23/07/2015	Polva	POLVA	GII-CVR1	
Est15/WB-RAPLA-1	TA1512165	EWB	23/07/2015	Kehtna	RAPLA	GII-CVR1	
Est15/DP-VALGA2	TA1512247	DP	25/07/2015	Viljandi	VILJANDI	GII-CVR1	
Est15/DP-JARVA1	TA1512503	DP	27/07/2015	Roosna-Alliku	JARVA	GII-CVR1	
Est15/WB-JARVA1	TA1513642	EWB	27/07/2015	Koeru	JARVA	GII-CVR1	
Est15/WB-POLVA-2	TA1514523	EWB	27/07/2015	Veriora	POLVA	GII-CVR1	
Est15/DP-Tartu1	TA1512410	DP	28/07/2015	Puhja	TARTU	GII-CVR1	
Est15/DP-Tartu2	TA1512627	DP	28/07/2015	Puhja	TARTU	GII-CVR1	
Est15/DP-VALGA3	TA1512551	DP	29/07/2015	Taheva	Valga	GII-CVR1	

Est15/DP-VIRU1	TA1512708	DP	01/08/2015	Rakke	LAANE-VIRU	GII-CVR1	
Est15/DP-Jogeva1	TA1513103	DP	04/08/2015	Puurmani	JOGEVA	GII-CVR1	
Est15/DP-Vil4	TA1512892	DP	04/08/2015	Tarvastu	VILJANDI	GII-CVR1	
Est15/WB-Tartu4	TA1514125[4]	EWB	04/08/2015	Rannu	TARTU	GII-CVR2	MT647529
Est15/DP-VORU1	TA1513204[2]	DP	07/08/2015	Haanja	VORU	GII-CVR1	
Est15/DP-VORU2	TA1513212[2]	DP	07/08/2015	Haanja	VORU	GII-CVR1	
Est15/DP-Vil5	TA1513456[1]	DP	08/08/2015	Tarvastu	VILJANDI	GII-CVR1	
Est15/WB-Tartu5	TA1513353	EWB	11/08/2015	Rannu	TARTU	GII-CVR1	
Est15/WB-Parnu2	TA1513669	EWB	14/08/2015	Saarde	PARNU	GII-CVR1	
Est15/WB-JARVA2	TA1513642	EWB	15/08/2015	Turi	JARVA	GII-CVR1	
Est15/DP-VALGA4	TA1514046[3]	DP	17/08/2015	Hummuli	VALGA	GII-CVR1	
Est15/DP-VALGA5	TA1514047[3]	DP	17/08/2015	Hummuli	VALGA	GII-CVR1	
Est15/WB-Tartu6	TA1513675	EWB	17/08/2015	Konguta	TARTU	GII-CVR1	
Est15/WB-Tartu7	TA1513676	EWB	17/08/2015	Puhja	TARTU	GII-CVR1	
Est15/DP-Jogeva2	TA15-13884	DP	19/08/2015	Puurmani	JOGEVA	GII-CVR1	
Est15/DP-VALGA6	TA1514044	DP	21/08/2015	Tõlliste	VALGA	GII-CVR1	
Est15/DP-VALGA7	TA1514044	DP	21/08/2015	Tõlliste	VALGA	GII-CVR1	
Est15/DP-Tartu3	TA1514031[4]	DP	23/08/2015	Puhja	TARTU	GII-CVR1	
Est15/DP-Tartu4	TA1514125[4]	DP	23/08/2015	Puhja	TARTU	GII-CVR1	
Est15/DP-Tartu5	TA1514125[4]	DP	23/08/2015	Puhja	TARTU	GII-CVR1	
Est15/WB-Tartu8	TA1514201	EWB	24/08/2015	Kambja	TARTU	GII-CVR1	
Est15/WB-Tartu10	TA1514570	EWB	27/08/2015	Peipsiaare	TARTU	GII-CVR1	
Est15/WB-Tartu9	TA1514571	EWB	27/08/2015	Konguta	TARTU	GII-CVR1	
Est15/WB-Jogeva1	TA1514623	EWB	01/09/2015	Poltsamaa	JOGEVA	GII-CVR1	
Est15/WB-Jogeva2	TA1514689	EWB	01/09/2015	Poltsamaa	JOGEVA	GII-CVR1	
Est15/WB-Tartu11	TA1515910	EWB	15/09/2015	Puhja	TARTU	GII-CVR1	
Est15/DP2-VORU3	TA1516596-1	DP	19/09/2015	Haanja	VORU	GII-CVR1	
Est15/DP3-VORU3	TA1516596-2	DP	19/09/2015	Haanja parish	VORU	GII-CVR1	
Est15/WB-Jogeva3	TA1517888	EWB	03/10/2015	Tabivere	JOGEVA	GII-CVR1	
Est15/WB-POLVA-3	TA1518521	EWB	12/10/2015	Valgjarve	POLVA	GII-CVR1	
Est15/WB-Tartu12	TA1518533	EWB	12/10/2015	Kambja	TARTU	GII-CVR1	
Est15/WB-Tartu13	TA1521483	EWB	04/11/2015	Nõo	TARTU	GII-CVR1	

Est15/WB-Tartu14	TA1521528	EWB	04/11/2015	Konguta	TARTU	GII-CVR2	MT647530
Est15/WB-IDA-3	TA1524606	EWB	05/11/2015	Lohusuu	IDA-VIRU	GII-CVR1	
Est15/WB-JARVA3	TA1522201	EWB	13/11/2015	Koeru	JARVA	GII-CVR1	
Est15/WB-VIRU1	TA1522535	EWB	15/11/2015	Maarja	LAANE-VIRU	GII-CVR1	
Est15/WB-Tartu15	TA1522376	EWB	15/11/2015	Alatskivi	TARTU	GII-CVR1	
Est15/WB-JARVA4	TA1522446	EWB	17/11/2015	Koeru	JARVA	GII-CVR1	
Est15/WB-Jogeva4	TA1522419	EWB	17/11/2015	Pajusi	JOGEVA	GII-CVR1	
Est15/WB-JARVA5	TA1522649	EWB	18/11/2015	Koeru	JARVA	GII-CVR1	
Est15/WB-Tartu16	TA1523104	EWB	25/11/2015	Kambja	TARTU	GII-CVR1	
Est15/WB-POLVA-4	TA1525192	EWB	15/12/2015	Polva	POLVA	GII-CVR1	
Est15/WB-VIRU2	TA1525592	EWB	17/12/2015	Maarja	LAANE-VIRU	GII-CVR1	
Est15/WB-Parnu3	TA1525910	EWB	19/12/2015	Vandra	PARNU	GII-CVR1	
Est15/WB-RAPLA-2	TA1526390	EWB	27/12/2015	Kehtna	RAPLA	GII-CVR1	
Est15/WB-VIRU3	TA1526399	EWB	30/12/2015	Tapa	LAANE-VIRU	GII-CVR1	
Est16/WB-VIRU4	TA1600239	EWB	08/01/2016	Laekvere	LAANE-VIRU	GII-CVR1	
Est16/WB-Tartu17	TA1600798	EWB	10/01/2016	Rannu	TARTU	GII-CVR2	MT647531
Est16/WB-TARTU25	TA1600745	EWB	12/01/2016	Meeksi	TARTU	GII-CVR1	
Est16/WB-IDA-4	TA1626835	EWB	16/01/2016	Lüganuse	IDA-VIRU	GII-CVR1	
Est16/WB-RAPLA-3	TA1600997	EWB	17/01/2016	Kehtna	RAPLA	GII-CVR1	
Est16/WB-JOGEVA5	TA1603353	EWB	01/02/2016	Puurmani	JOGEVA	GII-CVR1	
Est16/WB-POLVA-5	TA1604026	EWB	05/02/2016	Varska	POLVA	GII-CVR1	
Est16/WB-Parnu4	TA1604196	EWB	09/02/2016	Vandra	PARNU	GII-CVR1	
Est16/WB-VIL23	TA1604871	EWB	16/02/2016	Suure-Jaani	Viljandi	GII-CVR1	
Est16/WB-VIRU5	TA1616618	EWB	20/02/2016	Kadrina	LAANE-VIRU	GII-CVR1	
Est16/WB-Tartu18	TA1605469	EWB	20/02/2016	Rannu	TARTU	GII-CVR2	MT647532
Est16/WB-TARTU26	TA1606075	EWB	26/02/2016	Laeva	TARTU	GII-CVR1	
Est16/WB-Tartu19	TA1607606	EWB	13/03/2016	Konguta	TARTU	GII-CVR2	MT647533
Est16/WB-Tartu20	TA1609189	EWB	05/04/2016	Tähtvere	TARTU	GII-CVR1	
Est16/WB-Tartu21	TA1609759	EWB	14/04/2016	Nõo	TARTU	GII-CVR1	
Est16/WB-Tartu22	TA1610184	EWB	21/04/2016	Tartu	TARTU	GII-CVR1	
Est16/WB-Valga12	TA1614663	EWB	06/06/2016	Palupera	VALGA	GII-CVR1	

Est16/WB-JOGEVA6	TA1612790	EWB	07/06/2016	Jõgeva	JOGEVA	GII-CVR1
Est16/WB-JOGEVA7	TA1612933	EWB	07/06/2016	Saare	JOGEVA	GII-CVR1
Est16/WB-RAPLA6	TA1613805	EWB	21/06/2016	Raikküla	RAPLA	GII-CVR1
Est16/DP-JOGEVA3	TA1614387	DP	30/06/2016	Puurmani	JOGEVA	GII-CVR1
Est16/WB-HARJU1	TA1614202	EWB	30/06/2016	Jõelähtme	HARJU	GII-CVR1
Est16/DP-JOGEVA4	TA1614483	DP	02/07/2016	Poltsamaa	JOGEVA	GII-CVR1
Est16/DP-JOGEVA5	TA1614493	DP	03/07/2016	Saare	JOGEVA	GII-CVR1
Est16/WB-Parnu6	TA1614477	EWB	04/07/2016	Tori	PARNU	GII-CVR1
Est16/WB-VORU11	TA1614636	EWB	06/07/2016	Urvaste	VORU	GII-CVR1
Est16/WB-JARVA6	TA1614701	EWB	07/07/2016	Türi	JARVA	GII-CVR1
Est16/WB-JOGEVA8	TA1614720	EWB	07/07/2016	Puurmani	JOGEVA	GII-CVR1
Est16/DP-JARVA2	TA1614890	DP	10/07/2016	TURI	JARVA	GII-CVR1
Est16/DP-JARVA3	TA1615001-5	DP	11/07/2016	TURI	JARVA	GII-CVR1
Est16/WB-POLVA6	TA1614800	EWB	11/07/2016	Mikitamäe	POLVA	GII-CVR1
Est16/WB-Tartu23	TA1614801	EWB	11/07/2016	Rõngu	TARTU	GII-CVR1
Est16/WB-Tartu24	TA1614991	EWB	12/07/2016	Rõngu	TARTU	GII-CVR1
Est16/DP-VIRU2	TA1615500	DP	18/07/2016	Kadrina	LAANE-VIRU	GII-CVR1
Est16/WB-JOGEVA9	TA1615454	EWB	20/07/2016	Saare	JOGEVA	GII-CVR1
Est16/WB-HARJU2	TA1616524	EWB	09/08/2016	Kiili	HARJU	GII-CVR1
Est16/WB-VIRU5	TA1616618	EWB	10/08/2016	Sagadi	LAANE-VIRU	GII-CVR1
Est16/WB-VIRU6	TA1616693	EWB	11/08/2016	Kadrina	LAANE-VIRU	GII-CVR1
Est16/WB-RAPLA-4	TA1616879	EWB	16/08/2016	Marjamaa	RAPLA	GII-CVR1
Est16/WB-HARJU3	TA1617225	EWB	22/08/2016	Kose	HARJU	GII-CVR1
Est16/DP-SAARE1	TA1617404-1	DP	23/08/2016	Valjala	SAARE	GII-CVR1
Est16/WB-RAPLA-5	TA1617462	EWB	25/08/2016	Marjamaa	RAPLA	GII-CVR1
Est16/WB-Parnu5	TA1617575	EWB	26/08/2016	Audru	PARNU	GII-CVR1
Est16/WB-SAARE1	TA1617569	EWB	26/08/2016	Pihtla	SAARE	GII-CVR1
Est16/DP-SAARE2	TA1617643	DP	31/08/2016	Laimjala	SAARE	GII-CVR1
Est16/WB-RAPLA7	TA1618660	EWB	13/09/2016	Kohila	RAPLA	GII-CVR1
Est16/WB-Valga13	TA1619069	EWB	20/09/2016	Õru	VALGA	GII-CVR1
Est16/WB-Saarema1	TA1619685	EWB	27/09/2016	Orissaare	SAARE	GII-CVR1

Est16/WB-VIL24	TA1621142	EWB	16/10/2016	Karksi	VILJANDI	GII-CVR1		
Est16/WB-Saarema2	TA1621477	EWB	19/10/2016	Valjala	SAARE	GII-CVR1		
Est16/WB-HARJU4	TA1621920	EWB	25/10/2016	Saku	HARJU	GII-CVR1		
Est16/WB-VIL25	TA1622401	EWB	01/11/2016	Kõpu	VILJANDI	GII-CVR1		
Est16/WB-RAPLA8	TA1623127	EWB	09/11/2016	Juuru	RAPLA	GII-CVR1		
Est16/WB-Saarema3	TA1623171	EWB	09/11/2016	Pihtla	SAARE	GII-CVR1		
Est16/WB-HARJU5	TA1624174	EWB	16/11/2016	Kernu	HARJU	GII-CVR1		
Est16/WB-LANE1	TA1623939	EWB	16/11/2016	Hanila	LAANE	GII-CVR1		
Est16/WB-Parnu7	TA1623942	EWB	16/11/2016	Halinga	PARNU	GII-CVR1		
Est16/WB-TARTU27	TA1624271	EWB	17/11/2016	Peipsiääre	TARTU	GII-CVR1		
Est16/WB-LANE2	TA1625158	EWB	28/11/2016	Kullamaa	LAANE	GII-CVR1		
Est16/WB-IDA-5	TA1625355	EWB	29/11/2016	Mäetaguse	IDA-VIRU	GII-CVR1		
Est16/WB-LANE3	TA1625439	EWB	30/11/2016	Hanila	LAANE	GII-CVR1		
Est16/WB-VIRU8	TA1625367	EWB	30/11/2016	Viru-Nigula	LAANE-VIRU	GII-CVR1		
Est16/WB-Saarema4	TA1625647	EWB	01/12/2016	Salme	SAARE	GII-CVR1		
Est16/WB-LANE4	TA1625728	EWB	02/12/2016	Lihula	LAANE	GII-CVR1		
Est16/WB-LANE5	TA1625761	EWB	02/12/2016	Lihula	LAANE	GII-CVR1		
Est16/WB-Parnu8	TA1625830	EWB	04/12/2016	Koonga	PARNU	GII-CVR1		
Est16/WB-Saarema5	TA1626711	EWB	13/12/2016	Lääne-Saare	SAARE	GII-CVR1		
Est16/WB-IDA4	TA1626835	EWB	14/12/2016	Lüganuse	IDA-VIRU	GII-CVR1		
Est16/WB-Saarema6	TA1626850	EWB	14/12/2016	Leisi	SAARE	GII-CVR1		
Est16/WB-VIRU9	TA1627241	EWB	21/12/2016	Sõmeru	LAANE-VIRU	GII-CVR1		
Est16/WB-Parnu9	TA1627788	EWB	30/12/2016	Varbla	PARNU	GII-CVR1		
Est17/WB-LANE6	TA1700178	EWB	04/01/2017	Laane-Nigula	LAANE	GII-CVR1		
Est17/WB-HARJU6	TA1700255	EWB	05/01/2017	Keila	HARJU	GII-CVR1/SNP1		MT647548
Est17/WB-Saarema7	TA1700871-1	EWB	11/01/2017	Laane-Saare	SAARE	GII-CVR1		
Est17/WB-Saarema8	TA1702697	EWB	01/02/2017	Kihelkonna	SAARE	GII-CVR1		
Est17/WB-TARTU28	TA1703393	EWB	08/02/2017	Tahtvere	TARTU	GII-CVR1		
Est17/WB-RAPLA9	TA1703427-3	EWB	13/02/2017	Juuru	RAPLA	GII-CVR1		
Est17/WB-HARJU7	TA1703582	EWB	13/02/2017	Padise	HARJU	GII-CVR1		
Est17/WB-LANE9	TA1704264	EWB	19/02/2017	Martna	LAANE	GII-CVR1		

Est17/WB-HARJU8	TA1704494	EWB	22/02/2017	Kiili	HARJU	GII-CVR1	
Est17/WB-RAPLA10	TA1704468-6	EWB	22/02/2017	Marjamaa	RAPLA	GII-CVR1	
Est17/WB-Saarema10	TA1704467	EWB	22/02/2017	Orissaare	SAARE	GII-CVR1	
Est17/WB-Saarema9	TA1705023	EWB	01/03/2017	Poide	SAARE	GII-CVR1	
Est17/WB-VIRU10	TA1705283-4	EWB	02/03/2017	Viru-Nigula	LAANE-VIRU	GII-CVR1	
Est17/WB-Parnu10	TA1706133-2	EWB	14/03/2017	Varbla	PARNU	GII-CVR1	
Est17/WB-Saarema11	TA1706131	EWB	14/03/2017	Valjala	SAARE	GII-CVR1	
Est17/WB-Saarema12	TA1707556	EWB	31/03/2017	Laimjala	SAARE	GII-CVR1	
Est17/WB-LANE7	TA1707844	EWB	06/04/2017	Viru-Nigula	LAANE	GII-CVR1	
Est17/WB-RAPLA11	TA1708959	EWB	25/04/2017	Kaiu	RAPLA	GII-CVR1	
Est17/WB-VIL27	TA1709316	EWB	02/05/2017	Viljandi	VILJANDI	GII-CVR1	
Est17/WB-LANE8	TA1710321	EWB	17/05/2017	Laekwere	LAANE-VIRU	GII-CVR1	
Est17/WB-Parnu11	TA1710544	EWB	22/05/2017	Tostamaa	PARNU	GII-CVR1/SNP1	MT647550
Est17/DP-Parnu1	TA1712030_8	DP	14/06/2017	Audru	PARNU	GII-CVR1/SNP1	MT647544
Est17/DP-Parnu2	TA1712030_10	DP	14/06/2017	Audru	PARNU	GII-CVR1/SNP1	MT647545
Est17/DP-Parnu3	TA1712030_2	DP	14/06/2017	Audru	PARNU	GII-CVR1/SNP1	MT647546
Est17/DP-Parnu4	TA1712030_6	DP	14/06/2017	Audru	PARNU	GII-CVR1/SNP1	MT647547
Est17/WB-Parnu12	TA1712190	EWB	16/06/2017	Audru	PARNU	GII-CVR1/SNP1	MT647551
Est17/WB-Parnu13	TA1713020	EWB	04/07/2017	Tostamaa	PARNU	GII-CVR1/SNP1	MT647552
Est17/WB-Saarema13	TA1713257	EWB	07/07/2017	Mustjlaa	SAARE	GII-CVR1	
Est17/DP-Saarema1	TA1713368_3	DP	11/07/2017	Orissaare	SAARE	GII-CVR1	
Est17/DP-Saarema2	TA1713368_6	DP	11/07/2017	Orissaare	SAARE	GII-CVR1	
Est17/DP-Saarema3	TA1713369_1	DP	11/07/2017	Orissaare	SAARE	GII-CVR1	
Est17/DP-Saarema4	TA1713369_4	DP	11/07/2017	Orissaare	SAARE	GII-CVR1	
Est17/WB-LANE10	TA1714365	EWB	22/07/2017	Laane-Nigula	LAANE	GII-CVR1	
Est17/WB-LANE11	TA1714357	EWB	22/07/2017	Ridala	LAANE	GII-CVR1	

Est17/WB-HARJU9	TA1714580	EWB	01/08/2017	Nissi	HARJU	GII-CVR1	
Est17/WB-LANE12	TA1715094	EWB	10/08/2017	Nova	LAANE	GII-CVR1/SNP1	MT647549
Est17/WB-LANE13	TA1716043	EWB	28/08/2017	Lihula	LAANE	GII-CVR1	
Est17/WB-Saarema14	TA1716218	EWB	30/08/2017	Laane-Saare	SAARE	GII-CVR1	
Est17/DP-LANE1	TA1717407_2	DP	19/09/2017	Laane-Nigula	LAANE	GII-CVR1	
Est17/DP-LANE2	TA1717407_4	DP	19/09/2017	Laane-Nigula	LAANE	GII-CVR1	
Est17/DP-LANE3	TA1717408_4	DP	19/09/2017	Laane-Nigula	LAANE	GII-CVR1	
Est17/WB-RAPLA13	TA1717539	EWB	20/09/2017	Rapla	RAPLA	GII-CVR1	
Est17/WB-LANE14	TA1717844	EWB	26/09/2017	Ridala	LAANE	GII-CVR1	
Est17/WB- VORU12	TA1722842	EWB	05/12/2017	Meremae	VORU	GII-CVR1	
Est17/WB-POLVA7	TA1723359	EWB	11/12/2017	Orava	POLVA	GII-CVR1	
Est17/WB-LANE15	TA1723414	EWB	12/12/2017	Vormsi	LAANE	GII-CVR1	
Est17/WB-Parnu14	TA1723411	EWB	12/12/2017	Tostamaa	PARNU	CVR1 SNP1	MT647553
Est17/WB-Saarema15	TA1723745	EWB	18/12/2017	Laane-Saare	SAARE	GII-CVR1	
Est18/WB-LANE16	TA1800294	EWB	09/01/2018	Laane-Nigula	LAANE	GII-CVR1	
Est18/WB-POLVA8	TA1800646	EWB	15/01/2018	Polva	POLVA	GII-CVR1	
Est18/WB-LANE17	TA1801323	EWB	25/01/2018	Laane-Nigula	LAANE	GII-CVR1	
Est18/WB-Saarema16	TA1801467	EWB	26/01/2018	Saaremaa	SAARE	GII-CVR1	
Est18/WB-HARJU10	TA1801610	EWB	30/01/2018	Lääne-Harju	HARJU	GII-CVR1	
Est17/WB-RAPLA12	TA1712189	EWB	19/06/2017	Vigala	RAPLA	GII-CVR1	
Est15/WB-Valga14	TA1505434	EWB	24/03/2015	Puka	VALGA	GII-CVR1	
Est15/WB-Valga15	TA1506497	EWB	14/04/2015	Puka	VALGA	GII-CVR1	
Est15/WB-Tartu28	TA1506686	EWB	16/04/2015	Rannu	TARTU	GII-CVR1	
Est15/WB-Tartu29	TA1507866_2	EWB	08/05/2015	Rõngu	TARTU	GII-CVR1	
Est15/WB-Valga16	TA1508135	EWB	13/05/2015	Hummuli	VALGA	GII-CVR1	
Est15/WB-Voru13	TA1510114	EWB	16/06/2015	Rõuge	VÕRU	GII-CVR1	
Est15/WB-Valga17	TA1510520	EWB	22/06/2015	Sangaste	VALGA	GII-CVR1	
Est15/WB-Valga18	TA1511212	EWB	07/07/2015	Karula	VALGA	GII-CVR1	
Est15/WB-Voru14	TA1511419	EWB	10/07/2015	Varstu	VÕRU	GII-CVR1	
Est15/WB-Tartu30	TA1511512	EWB	13/07/2015	Rannu	TARTU	GII-CVR2	MT647536
Est15/WB-Tartu31	TA1511809_2	EWB	17/07/2015	Rannu	TARTU	GII-CVR2	MT647537

Est15/WB-Tartu32	TA1511908	EWB	21/07/2015	Rannu	TARTU	GII-CVR1	
Est15/WB-Vil28	TA1511937	EWB	21/07/2015	Viljandi	VILJANDI	GII-CVR1	
Est15/WB-Tartu33	TA1512438_3	EWB	29/07/2015	Konguta	TARTU	GII-CVR2	MT647538
Est15/WB-Vil29	TA1512572_3	EWB	30/07/2015	Viljandi	VILJANDI	GII-CVR1	
Est15/WB-Valga19	TA1512765_1	EWB	04/08/2015	Taheva	VALGA	GII-CVR1	
Est15/WB-Valga20	TA1513033	EWB	07/08/2015	Taheva	VALGA	GII-CVR1	
Est15/WB-Jarva7	TA1513301	EWB	12/08/2015	Albu	JÄRVA	GII-CVR1	
Est15/WB-Tartu34	TA1514210	EWB	26/08/2015	Konguta	TARTU	GII-CVR2	MT647539
Est15/WB-Voru15	TA1514418	EWB	27/08/2015	Haanja	VÕRU	GII-CVR1	
Est15/WB-Tartu35	TA1515283	EWB	08/09/2015	Konguta	TARTU	GII-CVR1	
Est15/WB-Tartu36	TA1515427	EWB	09/09/2015	Konguta	TARTU	GII-CVR1	
Est15/WB-VIRU11	TA1516103	EWB	16/09/2015	Rakke	LAANE-VIRU	GII-CVR1	
Est15/WB-VIRU12	TA1516819	EWB	23/09/2015	Väike-Maarja	LAANE-VIRU	GII-CVR1	
Est15/WB-Jarva8	TA1517208	EWB	30/09/2015	Koeru	JARVA	GII-CVR1	
Est15/WB-Tartu37	TA1517240	EWB	29/09/2015	Tähtvere	TARTU	GII-CVR2	MT647534
Est15/WB-Voru16	TA1518477	EWB	12/10/2015	Varstu	VORU	GII-CVR1	
Est15/WB-Tartu38	TA1518507	EWB	12/10/2015	Kambja	TARTU	GII-CVR1	
Est15/WB-Voru17	TA1518604	EWB	14/10/2015	Haanja	VORU	GII-CVR1	
Est15/WB-VIRU13	TA1518673	EWB	13/10/2015	Rakke	LAANE-VIRU	GII-CVR1	
Est15/WB-Tartu39	TA1518740	EWB	14/10/2015	Konguta	TARTU	GII-CVR2	MT647535
Est15/WB-Tartu40	TA1518743_2	EWB	14/10/2015	Nõo	TARTU	GII-CVR2	MT647540
Est15/WB-Valga21	TA1519104	EWB	16/10/2015	Taheva	VALGA	GII-CVR1	
Est15/WB-Tartu41	TA1519210_1	EWB	19/10/2015	Rannu	TARTU	GII-CVR1	
Est15/WB-Tartu42	TA1519269_1	EWB	21/10/2015	Kambja	TARTU	GII-CVR1	
Est15/WB-Vil30	TA1520231	EWB	28/10/2015	Kolga-Jaani	VILJANDI	GII-CVR1	
Est15/WB-Jarva9	TA1521286	EWB	04/11/2015	Ambla	JARVA	GII-CVR1	
Est15/WB-Tartu43	TA1521684_5	EWB	08/11/2015	Kambja	TARTU	GII-CVR1	
Est15/WB-Tartu45	TA1522358_2	EWB	15/11/2015	Tähtvere	TARTU	GII-CVR2	MT647541
Est15/WB-Polva9	TA1522663	EWB	18/11/2015	Vastse-Kuuste	POLVA	GII-CVR1	
Est15/WB-Jogeva10	TA1522814	EWB	19/11/2015	Põltsamaa	JOGEVA	GII-CVR1	

Est15/WB-Tartu46	TA1522875	EWB	19/11/2015	Puhja	TARTU	GII-CVR1	
Est15/WB-Jarva10	TA1523929_6	EWB	30/11/2015	Roosna-Alliku	JARVA	GII-CVR1	
Est15/WB-Vil31	TA1524583	EWB	07/12/2015	Kolga-Jaani	VILJANDI	GII-CVR1	
Est15/WB-Tartu44	TA1525164	EWB	14/11/2015	Puhja	TARTU	GII-CVR1	
Est15/WB-Jogeva11	TA1525990	EWB	22/12/2015	Palamuse	JOGEVA	GII-CVR1	
Est15/WB-Tartu47	TA1526171	EWB	28/12/2015	Nõo	TARTU	GII-CVR2	MT647542
Est16/WB-Jogeva12	TA1600112_5	EWB	04/01/2016	Saare	JOGEVA	GII-CVR1	
Est16/WB-Jarva11	TA1600147	EWB	06/01/2016	Türi	JARVA	GII-CVR1	
Est16/WB-Tartu49	TA1600717_5	EWB	11/01/2016	Kambja	TARTU	GII-CVR1	
Est16/WB-Valga22	TA1603456	EWB	02/02/2016	Tõlliste	VALGA	GII-CVR1	
Est16/WB-Jarva12	TA1604381	EWB	10/02/2016	Roosna-Alliku	JARVA	GII-CVR1	
Est16/WB-Tartu51	TA1604530_2	EWB	11/02/2016	Laeva	TARTU	GII-CVR1	
Est16/WB-Tartu53	TA1604754	EWB	15/02/2016	Konguta	TARTU	GII-CVR1	
Est16/WB-Jogeva13	TA1605518	EWB	22/02/2016	Põltsamaa	JOGEVA	GII-CVR1	
Est16/WB-Jarva13	TA1606220	EWB	28/02/2016	Türi	JARVA	GII-CVR1	
Est16/WB-Jogeva14	TA1607112	EWB	08/03/2016	Puurmani	JOGEVA	GII-CVR1	
Est16/WB-Jogeva15	TA1608556_5	EWB	24/03/2016	Saare	JOGEVA	GII-CVR1	
Est16/WB-Jogeva16	TA1609533	EWB	11/04/2016	Põltsamaa	JOGEVA	GII-CVR1	
Est16/WB-Jogeva18	TA1612163	EWB	26/05/2016	Tabivere	JOGEVA	GII-CVR1	
Est16/WB-VIRU14	TA1613124	EWB	09/06/2016	Kadrina	LAANE-VIRU	GII-CVR1	
Est16/WB-Jogeva19	TA1613235_6	EWB	13/06/2016	Saare	JOGEVA	GII-CVR1	
Est16/WB-Jarva14	TA1614249_1-4	EWB	30/06/2016	Imavere	JARVA	GII-CVR1	
Est16/WB-Jogeva20	TA1614901	EWB	12/07/2016	Põltsamaa	JOGEVA	GII-CVR1	
Est16/WB-Vil33	TA1615022	EWB	13/07/2016	Kolga-Jaani	VILJANDI	GII-CVR1	
Est16/WB-VIRU15	TA1615809_5	EWB	26/07/2016	Kadrina	LAANE-VIRU	GII-CVR1	
Est16/WB-VIRU16	TA1621783	EWB	23/10/2016	Kadrina	LAANE-VIRU	GII-CVR1	
Est16/WB-Vil34	TA1622985	EWB	06/11/2016	Tarvastu	VILJANDI	GII-CVR1	
Est17/WB-Saarema17	TA1700644_2	EWB	11/01/2017	Laimjala	SAARE	GII-CVR1	

Est17/WB-Saarema18	TA1701333	EWB	16/01/2017	Laimjala	SAARE	GII-CVR1	
Est17/WB-Parnu16	TA1704272_1	EWB	20/02/2017	Tõstamaa	PARNU	GII-CVR1/SNP1	MT647558
Est17/WB-Tartu62	TA1704832_2	EWB	27/02/2017	Tähtvere	TARTU	GII-CVR1	
Est17/WB-Tartu63	TA1704834_1	EWB	27/02/2017	Tähtvere	TARTU	GII-CVR1	
Est17/WB-Tartu65	TA1707999	EWB	10/04/2017	Tähtvere	TARTU	GII-CVR1	
Est17/WB-Parnu17	TA1710827	EWB	25/05/2017	Audru	PARNU	GII-CVR1/SNP1	MT647560
Est17/WB-Harju12	TA1706701	EWB	21/03/2017	Keila	HARJU	GII-CVR1/SNP1	MT647559
Est17/WB-Harju13	TA1713018	EWB	04/07/2017	Keila	HARJU	GII-CVR1/SNP1	MT647563
Est17/WB-Harju14	TA1713645	EWB	17/07/2017	Nissi	HARJU	GII-CVR1	
Est16/WB-LANE23	TA1626833	EWB	14/12/2016	Hanila	LAANE	GII-CVR1/SNP1	MT647555
Est16/WB-LANE22	TA1625439_2	EWB	30/11/2016	Hanila	LAANE	GII-CVR1/SNP1	MT647554
Est16/WB-LANE21	TA1623456	EWB	10/11/2016	Hanila	LAANE	GII-CVR1	
Est17/WB-LANE27	TA1702052	EWB	24/01/2017	Hanila	LAANE	GII-CVR1/SNP1	MT647557
Est17/WB-LANE26	TA1701015	EWB	12/01/2017	Lihula	LAANE	GII-CVR1	
Est17/WB-Tartu64	TA1705200	EWB	01/03/2017	Tähtvere	TARTU	GII-CVR1	
Est17/WB-Tartu61	TA1703558	EWB	13/02/2017	Tähtvere	TARTU	GII-CVR1	
Est16/WB-Tartu60	TA1615605	EWB	25/07/2016	Tartu	TARTU	GII-CVR1	
Est16/WB-Tartu50	TA1600814	EWB	13/01/2016	Nõo	TARTU	GII-CVR1	
Est16/WB-Tartu58	TA1611282_1	EWB	10/05/2016	Tähtvere	TARTU	GII-CVR1	
Est16/WB-Tartu57	TA1610151_2	EWB	21/04/2016	Tähtvere	TARTU	GII-CVR1	
Est16/WB-Tartu56	TA1608687	EWB	29/03/2016	Tähtvere	TARTU	GII-CVR2	MT647543
Est16/WB-Tartu48	TA1600640	EWB	08/01/2016	Tähtvere	TARTU	GII-CVR1	
Est16/WB-Tartu55	TA1608272_3	EWB	22/03/2016	Tähtvere	TARTU	GII-CVR1	
Est16/WB-Tartu54	TA1607132_1	EWB	08/03/2016	Kambja	TARTU	GII-CVR1	
Est16/WB-Tartu52	TA1604531_1	EWB	11/02/2016	Kambja	TARTU	GII-CVR1	
Est16/WB-Vil32	TA1607069	EWB	07/03/2016	Kolga-Jaani	VILJANDI	GII-CVR1	
Est16/WB-Tartu59	TA1614793	EWB	11/07/2016	Laeva	TARTU	GII-CVR1	
Est16/WB-Jogeva17	TA1610134	EWB	21/04/2016	Puurmani	JOGEVA	GII-CVR1	

Est17/WB-Parnu19	TA1712641	EWB	27/06/2017	Tõstamaa	PARNU	GII-CVR1/SNP1	MT647562
Est17/WB-Parnu20	TA1714358	EWB	27/07/2017	Tõstamaa	PARNU	GII-CVR1/SNP1	MT647564
Est17/WB-Parnu18	TA1712122_2	EWB	16/06/2017	Varbla	PARNU	GII-CVR1/SNP1	MT647561
Est17/WB-Parnu15	TA1701538_2	EWB	18/01/2017	Varbla	PARNU	GII-CVR1/SNP1	MT647556
Est17/WB-Parnu21	TA1719235	EWB	17/10/2017	Koonga	PARNU	GII-CVR1	
Est16/WB-LANE18	TA1620717	EWB	11/10/2016	Hanila	LAANE	GII-CVR1	
Est16/WB-LANE20	TA1623112	EWB	08/11/2016	Lihula	LAANE	GII-CVR1	
Est16/WB-LANE24	TA1627151	EWB	19/12/2016	Lihula	LAANE	GII-CVR1	
Est16/WB-LANE19	TA1620794_4	EWB	12/10/2016	Kullamaa	LAANE	GII-CVR1	
Est16/WB-LANE25	TA1627152	EWB	19/12/2016	Kullamaa	LAANE	GII-CVR1	
Est16/WB-Harju11	TA1625142	EWB	27/11/2016	Nissi	HARJU	GII-CVR1	
Est17/WB-Harju17	TA1722299	EWB	28/11/2017	Keila	HARJU	GII-CVR1	
Est17/WB-Harju15	TA1714142	EWB	25/07/2017	Nissi	HARJU	GII-CVR1	
Est17/WB-Harju16	TA1715175	EWB	10/08/2017	Padise	HARJU	GII-CVR1	
Est17/WB-Harju18	TA1722285_4	EWB	28/11/2017	Padise	HARJU	GII-CVR1	
Est18/WB-Harju19	TA1801610_1	EWB	30/01/2018	Lääne-Harju	HARJU	GII-CVR1	
Est17/WB-LANE31	TA1723397	EWB	12/12/2017	Noarootsi	LAANE	GII-CVR1/SNP1	MT647565
Est17/WB-LANE30	TA1720617	EWB	03/11/2017	Nõva	LAANE	GII-CVR1	
Est17/WB-LANE29	TA1718144	EWB	29/09/2017	Lääne-Nigula	LAANE	GII-CVR1	
Est17/WB-LANE28	TA1715100	EWB	09/08/2017	Lääne-Nigula	LAANE	GII-CVR1	
Est17/WB-Rapla15	TA1718024	EWB	27/09/2017	Vigala	RAPLA	GII-CVR1	
Est17/WB-Rapla14	TA1715789	EWB	22/08/2017	Märjamaa	RAPLA	GII-CVR1	
Est17/WB-Polva10	TA1721466_4	EWB	14/11/2017	Põlva	PÕLVA	GII-CVR1	
Est17/WB-Polva12	TA1724138_3	EWB	22/12/2017	Räpina	PÕLVA	GII-CVR1	
Est17/WB-Voru18	TA1722842	EWB	05/12/2017	Meremäe	VORU	GII-CVR1	
Est17/WB-Polva11	TA1723359	EWB	11/12/2017	Orava	POLVA	GII-CVR1	
Est16/WB-VIRU18	TA1706146	EWB	14/03/2017	Viru-Nigula	LAANE-VIRU	GII-CVR1	

Est16/WB-VIRU17	TA1702046	EWB	24/01/2017	Vinni	LAANE-VIRU	GII-CVR1	
Est17/WB-IDA6	TA1701394	EWB	17/01/2017	Lüganuse	IDA-VIRU	GII-CVR1	
Est17/WB-Saarema21	TA1713258	EWB	10/07/2017	Pihtla	SAARE	GII-CVR1	
Est17/WB-Saarema22	TA1713289	EWB	10/07/2017	Orissaare	SAARE	GII-CVR1	
Est17/WB-Saarema20	TA1709178	EWB	27/04/2017	Valjala	SAARE	GII-CVR1	
Est17/WB-Saarema19	TA1705248	EWB	03/03/2017	Valjala	SAARE	GII-CVR1	
Est18/WB-Tartu66	TA18-02001	EWB	05/02/2018	Tartu	TARTU	GII-CVR1	
Est18/WB-IDA7	TA1821394	EWB	18/12/2018	Narva-Jõesuu	IDA-VIRU	GII-CVR1	
Est19/WB-IDA8	TA1900448	EWB	08/01/2019	Narva-Jõesuu	IDA-VIRU	GII-CVR1	
Est17/DP-Parnu5	TA1712217_3	DP	16/06/2017	Audru	PARNU	GII-CVR1/SNP1	MT647566
Est17/DP-Parnu6	TA1712217_11	DP	16/06/2017	Audru	PARNU	GII-CVR1/SNP1	MT647567
Est17/DB-Saarema5	TA1713368_1	DP	11/07/2017	Orissaare	SAARE	GII-CVR1	
Est17/DB-Saarema6	TA1713369_1	DP	11/07/2017	Orissaare	SAARE	GII-CVR1	
Est17/DP-LANE4	TA1717407_885	DP	19/09/2017	Lääne-Nigula	LAANE	GII-CVR1	
Est17/DP-LANE5	TA1717408_3	DP	19/09/2017	Lääne-Nigula	LAANE	GII-CVR1	
